# Antihypertensive, antioxidant, and renal protective impact of integrated GJD with captopril in spontaneously hypertensive rats

**DOI:** 10.1038/s41598-023-38020-0

**Published:** 2023-07-06

**Authors:** Shadi A. D. Mohammed, Hanxing Liu, Salem Baldi, Yu Wang, Pingping Chen, Fang Lu, Shumin Liu

**Affiliations:** 1 Graduate School of Heilongjiang University of Chinese Medicine, Harbin, 150040 Heilongjiang China; 2 Research Center of Molecular Diagnostics and Sequencing, Axbio Biotechnology (Shenzhen) Co., Ltd., Shenzhen, 518057 Guangdong China; 3 Institute of Traditional Chinese Medicine, Heilongjiang University of Chinese Medicine, Harbin, 150040 Heilongjiang China; 4 School of Pharmacy, Lebanese International University, 18644 Sana’a, Yemen

**Keywords:** Molecular biology, Cardiology, Pathogenesis

## Abstract

Hypertension is the most prevalent chronic disease World-wide, and the leading preventable risk factor for cardiovascular disease (CVD). Few patients accomplish the objective of decreasing blood pressure and avoiding hypertensive target organ damage after treatments with antihypertensive agents which opens the door for other treatments, such as herbal-and antihypertensive combination therapy. Captopril (CAP), as a-pril which inhibits angiotensin converting enzyme has long been used in the management of hypertension and CVD. Gedan Jiangya Decoction (GJD) is known for antihypertensive effects in prior studies. The research is aimed to determine whether GJD in combination with captopril has antihypertensive, kidney protective, antioxidant, and vasoactive effects in spontaneously hypertensive rats (SHR). Regular measurements of systolic and diastolic blood pressure (SBP and DBP), and body weight were monitored weekly. H&E staining was utilized to examine histopathology. The combined effects were studied using ELISA, immunohistochemistry, and qRT-PCR. Significant reductions in SBP, DBP, aortic wall thickness, and improvement in renal tissue were observed following GJD + CAP treatment, with increased serum levels of NO, SOD, GSH-Px, and CAT and decreases in Ang II, ET-1, and MDA. Similarly, GJD + CAP treatment of SHR's significantly decreased ET-1 and AGTR1 mRNA and protein expression while increasing eNOS mRNA and protein expression in thoracic aorta and kidney tissue. In conclusion, the present investigation found that GJD + CAP treatment decreases SHR blood pressure, improves aorta remodeling and renal protection, and that this effect could be attributable, in part, due to antioxidant and vascular tone improvement.

## Introduction

Hypertension is among the main preventable risk factors for premature morbidity and mortality among humans in both the developing and developed worlds^1–3^. A study of 1,100,507 participants in low- and middle-income countries found that 192,441 (17.5%) had high blood pressure; 39.2% were diagnosed with hypertension, 29.9% were treated, and only 10.3% had hypertension under control^4^. In high-income countries, the rate of hypertension control is less than 25%^5^, whereas in low- and middle-income countries, it is only 8%^6^. Prolonged hypertensive state can cause hypertension-related target organ damage, which results in structural and functional impairment of critical internal organs such as the heart, kidneys, and brain^7^. These organs are primary targets of hypertension since they receive the majority of the blood flowing through the vascular system^8^. Of many mechanisms, reactive oxygen species (ROS) are produced primarily during hypertension development, by endothelial nitric oxide (eNOS) uncoupled to produce superoxide anion due to a combined reaction between reactive oxygen species and vasoconstriction factors such as endothelin-1 (ET-1) and angiotensin II (Ang II)^9^. The primary marker of hypertension is vascular damage and the etiology of hypertension-related target organ damage is the reduction in vasodilator nitric oxide and an elevation of oxidative stress, and vasoconstrictor factors ET-1 and Ang II^10–12^. Therefore, effective blood pressure-lowering intervention, along with antioxidants and a balance of vasoactive substances, has the potential for therapeutic intervention for preventing and treating hypertension-related cardiovascular disease^10,12,13^.

The development of hypertension is strongly influenced by various factors, including obesity, insulin resistance, high sodium intake, family history, and a sedentary lifestyle^14^. In the treatment of hypertension, there are multiple classes of drugs available. It is recommended to prioritize medications that have demonstrated effectiveness in reducing the risk of cardiovascular disease. Therefore, commonly used medications for hypertension treatment include angiotensin-converting enzyme inhibitors (ACEIs), angiotensin receptor blockers (ARBs), calcium channel blockers (CCBs), and thiazide diuretics^15^. Captopril, an ACE inhibitor, is widely utilized and has been proven to provide rapid antihypertensive effects, reduce peripheral vascular resistance, and offer protection against target organ damage associated with hypertension^16,17^.

In recent times, there has been growing evidence suggesting that Chinese herbal medicines can be effective in treating hypertension while maintaining a good safety profile^18,19^. Gedan Jiangya Decoction (GJD), a Chinese herbal formula (Patent Published No. CN114246896A) consists of six medicinal herbs, including *Achyranthes bidentata Blume, Prunella vulgaris, Eucommia ulmoides Oliv, Pueraria lobata Ohwi, Salvia miltiorrhiza,* and *Uncaria rhynchophylla*. Earlier studies have indicated that GJD (Chinese herbal medicine) can effectively reduce blood pressure in rats subjected to a high-salt diet. This blood pressure-lowering effect may be attributed to the decreased activation of the renin-angiotensin system^20^. In addition, our recently reported study revealed that the GJD could modulate cardiovascular inflammation and decreases blood pressure via regulating the NF-κB signal pathway in the spontaneously hypertensive rat^21^.

In the last decade, many studies have found that few patients have achieved the therapeutic goal of decreasing blood pressure from antihypertensive drugs and that herbal medicine (such as Chinese medicine) in combination therapy with antihypertensive drugs, is beneficial in decreasing blood pressure and preventing hypertensive related cardiovascular and kidney disease^22,23^, and the combination treatment has better antihypertensive efficacy than of monotherapy as it adds many mechanisms of action that block the different pathway of elevated blood pressure, in addition to offering greater protection to target organs than monotherapy and a lower risk of adverse effects^23–25^. The present study was designed to investigate the impact of GJD combined with captopril on blood pressure, oxidative stress, thoracic aorta remodeling, and kidney protection in spontaneously hypertensive rats.

## Results

### Arterial pressure measurement and body weight determination

**Figure 1:**
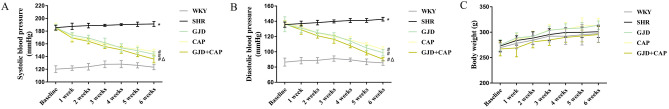
SBP, DBP, and body weight of each group's rats. (**A**) Systolic BP (**B**) Diastolic BP. (C) Body weight. The results are reported as means ± standard deviations. *P < 0.001 versus WKY, ^#^*P* < 0.001 versus SHR, and ^Δ^*P* < 0.001 versus CAP groups. (n = 8).

From (Fig. 1A,B), SBP and DBP were significantly higher in the SHR group (191.19 and 142.63 mmHg) than in the age-matched WKY group (123.55 and 85.51 mmHg) (all *P* < 0.001). Compared with the SHR group, significant decreases in SBP and DBP were observed in rats after 6 weeks of treatment with GJD (143.49 and 98.88 mmHg) and CAP (148.10 and 103.11 mmHg) (all *P* < 0.001), whereas there was no large difference between these groups. Similarly, SBP and DBP decreased significantly in the GJD + CAP group than in the SHR group (136.19 and 90.26 mmHg) (all *P* < 0.001). In addition, rats receiving GJD + CAP had the most significant reduction in SBP and DBP compared with the CAP group (all *P* < 0.001) (Fig. 1A,B). Throughout the observation period, our data showed no statistically significant differences (*P* > 0.05) in body weight between groups (Fig. 1C). (All data in Supplementary file 1).

### GJD and captopril combined effect on vasoconstriction factors Ang II, and ET-1 levels in serum

**Figure 2:**
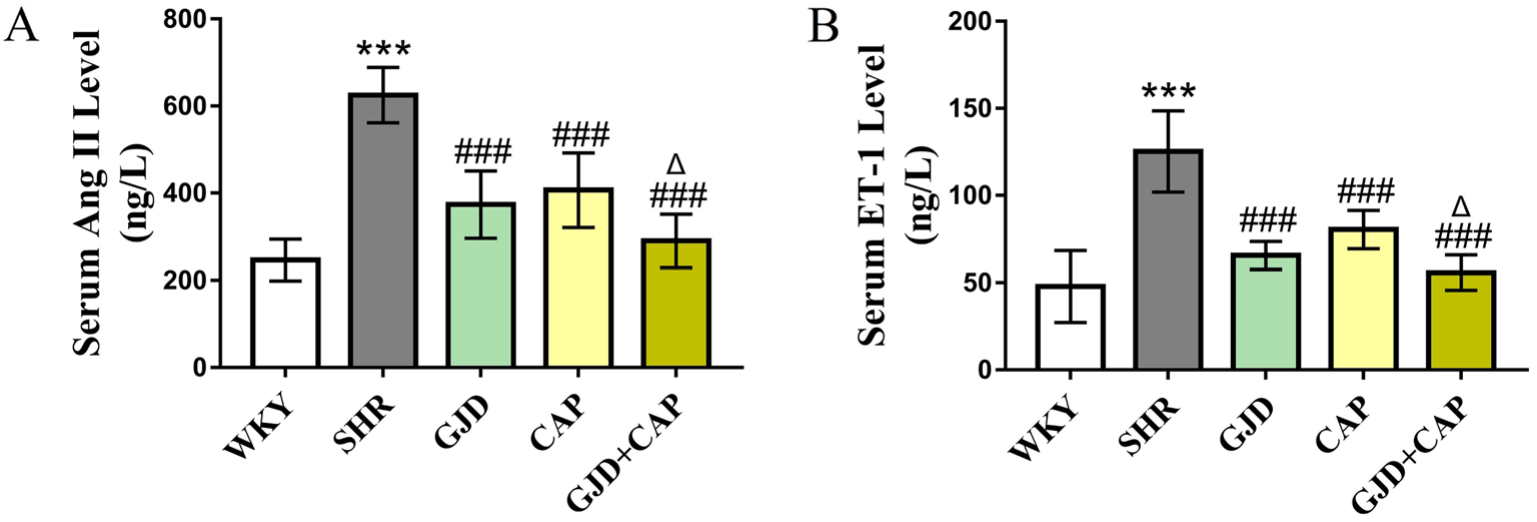
Serum levels of vasoconstriction factors Ang II and ET-1 of all rats in each group. (**A**) Ang II, (**B**) ET-1. The results are reported as means ± standard deviations. ***P < 0.001 compared the WKY, ^#^*P* < 0.05, ^##^*P* < 0.01, ^###^*P* < 0.001 versus SHR, and ^Δ^*P* < 0.05 versus CAP groups. (n = 8).

After 6 weeks of treatment, the SHR group had higher levels of the vasoconstrictor Ang II and ET-1 (625.3 and 125.3 ng/L) than the WKY group (246.6 and 47.8 ng/L) (all *P* < 0.001), whereas the levels of Ang II and ET-1 were significantly lower in the GJD, CAP, and GJD + CAP groups (Ang II 373.9, 406.9, and 290.5 ng/L; ET-1 65.6, 80.4, and 55.8 ng/L) than in the SHR group (all *P* < 0.001). In addition, the GJD + CAP group had significantly lower levels of Ang II and ET-1 than the CAP group (*P* = 0.01 and *P* = 0.03). (Fig. 2A,B).

### Expression of AGTR1, ET-1 and eNOS in thoracic aorta by Immunohistochemistry and qRT-PCR

**Figure 3:**
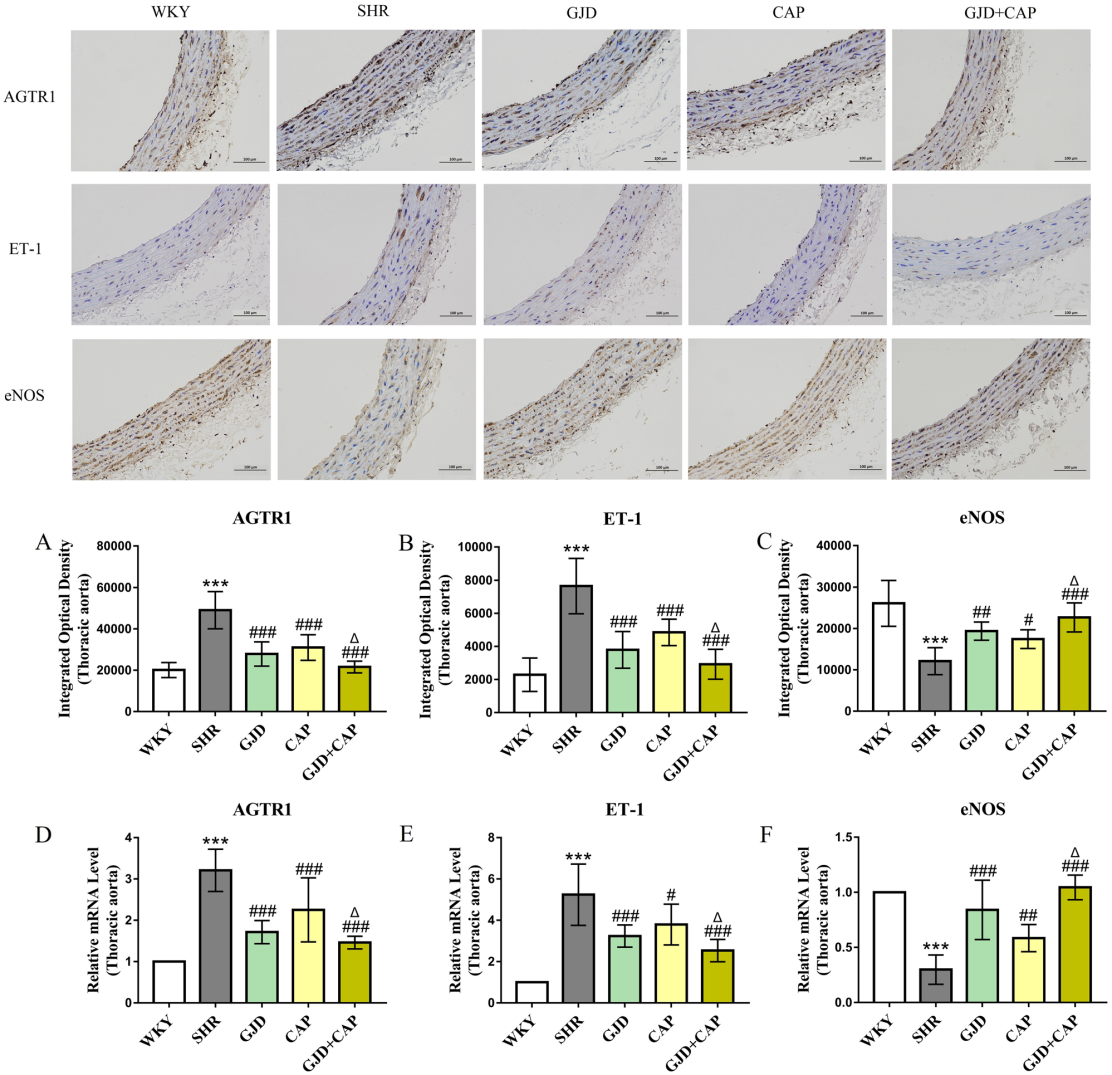
Immunohistochemistry and mRNA expression of AGTR1, ET-1, and eNOS in the thoracic aorta. (**A**) protein expressions of AGTR1, (**B**) protein expressions of ET-1, (**C**) protein expressions of eNOS, (**D**) mRNA expression of AGTR1, (**E**) mRNA expression of ET-1, and (**F**) mRNA expression of eNOS. (× 200 Magnification, scale bar: 100 μm). The results are reported as means ± standard deviations. ***P < 0.001 versus WKY, ^#^*P* < 0.05, ^##^*P* < 0.01, ^###^*P* < 0.001 versus SHR, and ^Δ^*P* < 0.05 versus CAP groups. (n = 8).

In the aorta, the immunohistochemical results are presented here as integrated optical density (IOD) values. AGTR1 and ET-1 expressions were significantly higher in the SHR group (49109 and 7656.41) than in the WKY group (20139 and 2290.94); meanwhile, eNOS expression was significantly reduced in the SHR group compared with the WKY group (12126.2 and 26101) (all *P* < 0.001), Fig. 3A–C. In contrast to the SHR group, the AGTR1 and ET-1 expression markedly decreased in GJD, CAP, and GJD + CAP groups (AGTR1 27885, 31036, and 21620; ET-1 3796.29, 4854.08, and 2924.48) (all *P* < 0.001), whereas eNOS expression dramatically increased in GJD, CAP, and GJD + CAP groups (19376.3, 17452.6, and 22689.3) (*P* = 0.002,* P* = 0.04, and *P* < 0.001), Fig. 3A–C. The GJD + CAP group demonstrated a significant reduction in AGTR1 and ET-1 and a substantial elevation in eNOS level than the CAP group (*P* = 0.02, *P* = 0.01, and *P* = 0.04).

The mRNA of AGTR1, ET-1, and eNOS was also examined using qRT-PCR. As shown in Fig. 3D–F, the SHR group's AGTR1 and ET-1 mRNA expression levels (3.21 and 5.24) increased dramatically more than the WKY group (1.00 and 1.00); meanwhile, eNOS mRNA expression level in SHR dramatically reduce more than WKY group (0.30 and 1.00) (all *P* < 0.001). The AGTR1 mRNA level was considerably lower in GJD, CAP, and GJD + CAP groups (1.72, 2.25, and 1.46) as compared to the SHR group (all *P* < 0.001), and the level of ET-1 mRNA was also dramatically reduced in GJD, CAP, and GJD + CAP groups (3.24, 3.80, and 2.54) (*P* < 0.001, *P* = 0.02, and *P* < 0.001), while the eNOS mRNA level significantly increased in GJD, CAP, and GJD + CAP groups (0.84, 0.59, and 1.04) (*P* < 0.001, *P* = 0.006, and *P* < 0.001). From Fig. 3D–F, the GJD + CAP group demonstrated a significant decrease in AGTR1 and ET-1 mRNA and a marked increase in eNOS mRNA level compared with the CAP group (*P* = 0.008, *P* = 0.05, and *P* < 0.001).

### Expression of AGTR1, ET-1 and eNOS in kidney by Immunohistochemistry and qRT-PCR

**Figure 4:**
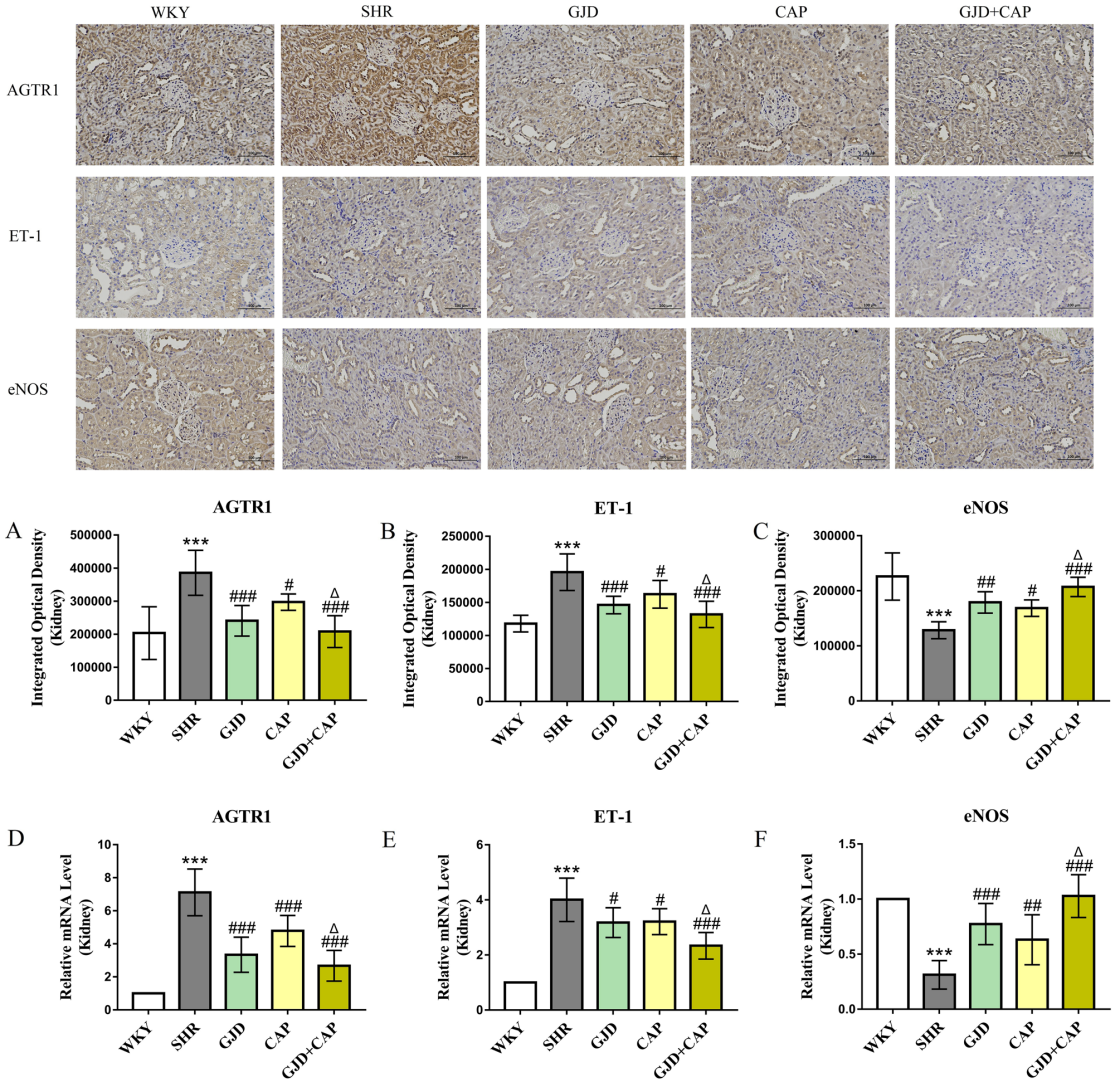
Immunohistochemistry and mRNA expression of AGTR1, ET-1, and eNOS in the kidney. (**A**) protein expressions of AGTR1, (**B**) protein expressions of ET-1, (**C**) protein expressions of eNOS, (**D**) mRNA expression of AGTR1, (**E**) mRNA expression of ET-1, and (**F**) mRNA expression of eNOS. (× 200 Magnification, scale bar: 100 μm). The results are reported as means ± standard deviations. ***P < 0.001 versus WKY, ^#^*P* < 0.05, ^##^*P* < 0.01, ^###^*P* < 0.001 versus SHR, and ^Δ^*P* < 0.05 versus CAP groups. (n = 8).

As demonstrated in Fig. 4A–C, the IOD values of AGTR1 and ET-1 expression in the kidney were significantly higher in the SHR group (385676 and 195664) than in the WKY group (203427.6 and 117899), whereas the expression of eNOS was significantly lower in SHR group compared to that of the WKY group (128498 and 226023) (all *P* < 0.001). As shown in Fig. 4A–C that when compared to the SHR group, AGTR1 (240698.13, 297121.87, and 207926) (*P* < 0.001, *P* = 0.03, and *P* < 0.001) and ET-1(146094, 162293, and 131873) (*P* < 0.001, *P* = 0.01, and *P* < 0.001) expressions were dramatically reduced in GJD, CAP, and GJD + CAP groups and expression of eNOS was significantly increased in GJD, CAP, and GJD + CAP groups (179001, 168597, and 207052) (*P* = 0.002, *P* = 0.02, and *P* < 0.001). Figure 4D–F, both AGTR1 and ET-1 expressions were significantly lower, and eNOS was significantly higher in the GJD + CAP group versus the CAP group (all *P* = 0.03).

The AGTR1, ET-1, and eNOS mRNA expressions were also determined using qRT-PCR. The AGTR1 and ET-1 mRNA levels were considerably higher in the SHR group (7.12 and 4.01) than in the WKY group (1.00 and 1.00); however, the eNOS mRNA level in SHR was significantly lower than that in the WKY group (0.31 and 1.00) (all *P* < 0.001). From Fig. 4D, the AGTR1 mRNA level was significantly reduced in GJD, CAP, and GJD + CAP groups (3.34, 4.79, and 2.67) (all *P* < 0.001) following treatment compared with the SHR group. The ET-1 level was considerably lower in GJD, CAP, and GJD + CAP groups (3.18, 3.21, and 2.34) than in the SHR group (*P* = 0.02, *P* = 0.03, and *P* < 0.001), Fig. 4E. In contrast to the SHR group, the mRNA expression of eNOS in GJD, CAP, and GJD + CAP groups was significantly increased (0.77, 0.63, and 1.03) (*P* < 0.001, *P* = 0.005, and *P* < 0.001), Fig. 4F. As shown in Fig. 4D–F, The GJD + CAP group showed markedly lower AGTR1 and ET-1 mRNA expression and substantially higher eNOS mRNA expression than the CAP group (*P* = 0.001, *P* = 0.02, and *P* < 0.001).

### GJD and captopril combined effect on oxidative stress

**Figure 5:**
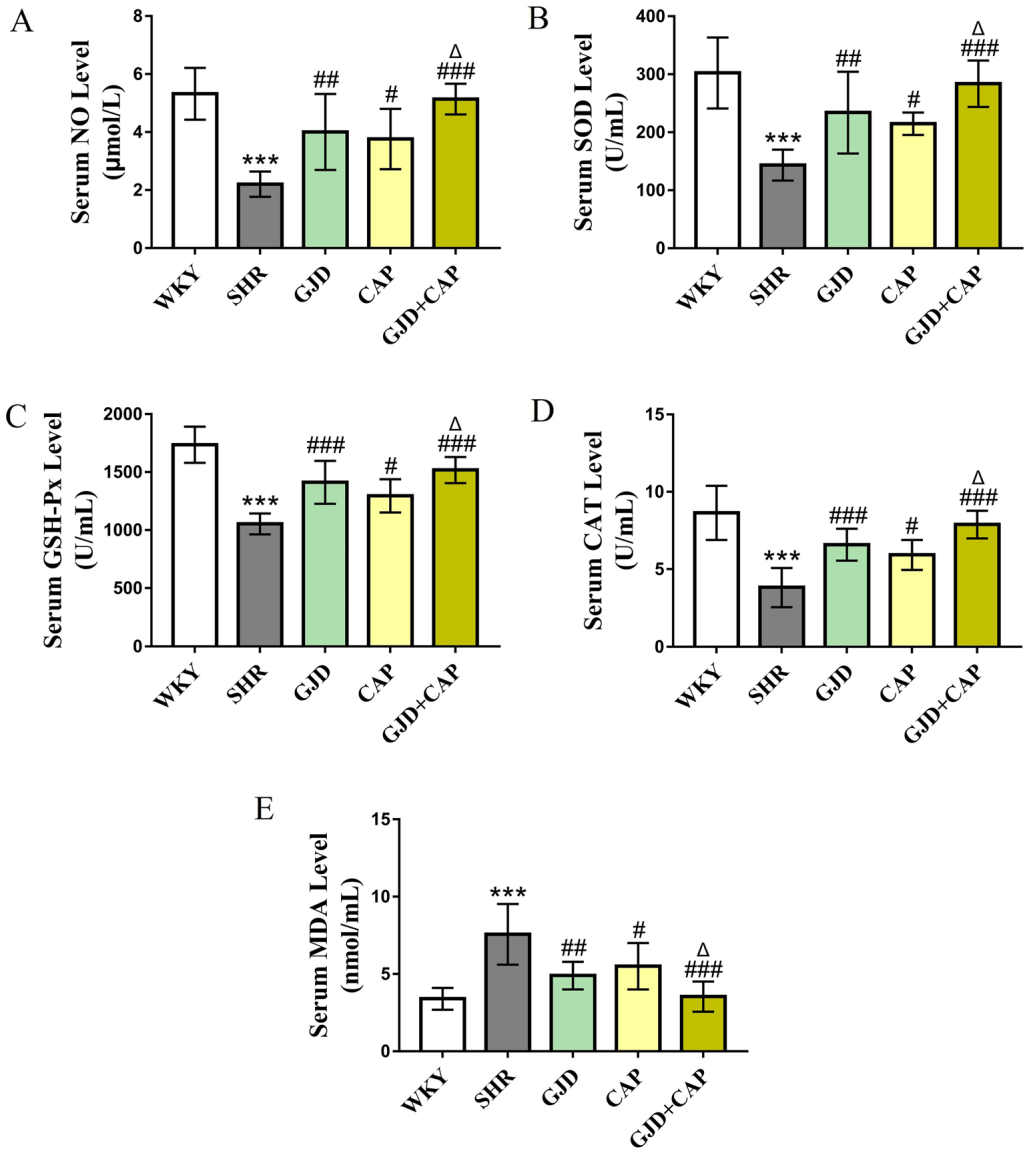
Serum level and activity of oxidative stress parameters of all rats in each group. (**A**) NO, (**B**) SOD, (**C**) GSH-Px, (**D**) CAT, (**E**) MDA. The results are reported as means ± standard deviations. ***P < 0.001 versus WKY, ^#^*P* < 0.05, ^##^*P* < 0.01, ^###^*P* < 0.001 versus SHR, ^Δ^*P* < 0.05 versus CAP groups. (n = 8).

As shown in Fig. 5A–E, SHR had significantly lower levels of nitric oxide (NO) (2.2* µmol*/L), superoxide dismutases (SOD) (143.4 U/ml), glutathione peroxidase (GSH-Px) (1054.6 U/ml), and catalase (CAT) (3.83 + 1 U/ml) and a significantly greater level of MDA (7.57 nmol/ml) than the age-matched WKY (5.47 µmol/L, 302.4 U/ml, 1736.3 U/ml, 8.64 U/ml, and 3.40 nmol/ml, respectively) (all *P* < 0.001). After 6 weeks of gavage treatment, NO level was significantly elevated in GJD, CAP, and GJD + CAP groups (4.01 µmol/L, 3.76 µmol/L, and 5.14 µmol/L) in comparison to SHR (*P* = 0.003, *P* = 0.01, and *P* < 0.001), Fig. 5A. The SOD serum level was significantly increased in GJD, CAP, and GJD + CAP groups (234.1, 214.9, and 284.0 U/ml) than that of the SHR group (*P* = 0.005, *P* = 0.04, and *P* < 0.001), Fig. 5B. In comparison to the SHR, the serum level of GSH-Px was also significantly improved in GJD, CAP, and GJD + CAP groups (1411.9, 1295.7, and 1518.9 U/ml) (*P* < 0.001, *P* = 0.01, and *P* < 0.001), Fig. 5C. After 6 weeks of treatment, the GJD, CAP, and GJD + CAP groups all had significantly greater CAT levels (6.59, 5.93, and 7.89 U/ml) compared to the SHR group (*P* < 0.001, *P* = 0.01, and *P* < 0.001), Fig. 5D. In contrast, the malondialdehyde (MDA) level in the rat's serum was reduced markedly in GJD, CAP, and GJD + CAP groups (4.90, 5.51, and 3.55 nmol/ml) in comparison to SHR (*P* = 0.002, *P* = 0.02, and *P* < 0.001), Fig. 5E. In contrast to the CAP group, the maximum increases in NO, SOD, GSH-Px, CAT, and minimum decreases in MDA, were observed in the rat's serum in the GJD + CAP group (*P* = 0.03, *P* = 0.05, *P* = 0.03, *P* = 0.02 and* P* = 0.03), Fig. 5A–E.

### Biochemicals parameters to assess liver and kidney function

As markers of hepatic and renal function, AST (*P* = 0.4073), ALT (*P* = 0.9552), TP (*P* = 0.085), BUN (*P* = 0.0273), CRE (*P* = 0.9699), DBIL (*P* = 0.0795), GGT (*P* = 0.6133), and TBA (*P* = 0.9348). The results of the one-way analysis ANOVA revealed that there was no significant difference in all liver and kidney function indices except BUN. However, Tukey's multiple comparison test showed that there was no significant difference in all liver and kidney function indices of each group in pairs (Table 1).

**Table 1:** Serum biochemical parameters in all groups.

Biochemical parameter	WKY	SHR	GJD	CAP	GJD + CAP
CRE (μmol/L)	46.74 ± 5.63	46.57 ± 8.42	48.1 ± 20.42	44.17 ± 10.72	44.8 ± 5.76
BUN (mmol/L)	6.96 ± 0.49	8.09 ± 0.9	7.46 ± 0.59	8.09 ± 0.65	8.05 ± 1.02
TP (g/L)	47.76 ± 5.74	54.76 ± 5.31	49.13 ± 2.69	50.8 ± 4.81	51.13 ± 4.15
AST (U/L)	174.97 ± 34.67	205.6 ± 33.28	184.17 ± 37.55	192.06 ± 19.02	191.86 ± 16.55
ALT (U/L)	64.28 ± 24.15	68.17 ± 11.32	70.15 ± 26.88	70.73 ± 12.8	70.98 ± 7.50
DBIL (μmol/L)	2.63 ± 0.29	3.25 ± 0.69	3.05 ± 0.29	3.03 ± 0.37	3.16 ± 0.28
TBA (μmol/L)	32.37 ± 11.92	37.08 ± 14.88	33.08 ± 9.27	35.02 ± 7.55	34.37 ± 8.58
GGT (U/L)	0.52 ± 0.22	0.75 ± 0.40	0.63 ± 0.28	0.74 ± 0.28	0.71 ± 0.30

### HE staining results

**Figure 6:**
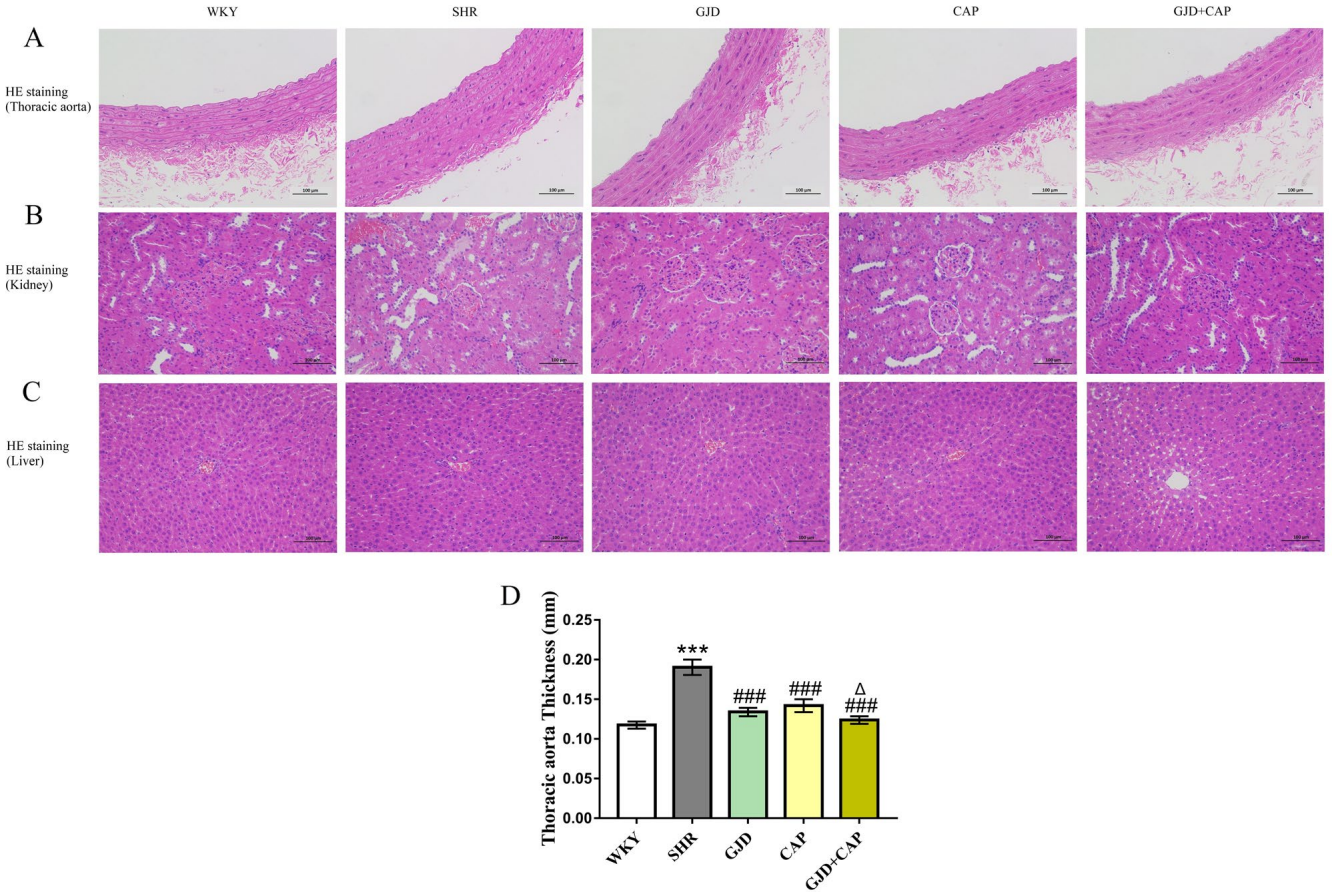
HE staining of thoracic aorta, kidney, and liver. (**A**) Thoracic aorta, (**B**) Kidney, (**C**) Liver, (**D**) Thoracic aorta thickness. (×200 Magnification, scale bar: 100 μm). The results are reported as means ± standard deviations. ***P < 0.001 versus WKY, ^###^*P* < 0.001 versus SHR, ^Δ^*P* < 0.05 versus CAP groups. (n = 8).

SHR displayed aorta thickening (0.190 mm), as shown by an increase in wall thickness when compared to age-matched WKY rats (0.118 mm) (*P* < 0.001). According to measurements of the thoracic aorta thickness, the progression of aortic hypertrophy was significantly attenuated in GJD, CAP, and GJD + CAP groups (0.134, 0.142, and 0.124 mm) than the SHR group (all *P* < 0.001), and thoracic aorta thickness was dramatically reduced in the GJD + CAP group than the CAP group (*P* < 0.001), Fig. 6A,D. The kidney tissue showed mild congestive dilation of glomerular capillaries in the model group, and no obvious abnormality was found in the other groups (The tissue staining was uniform, the glomerular morphology and structure were normal, the tubular epithelial cells were closely arranged, and no obvious inflammation was found), Fig. 6B. Mild hepatocyte degeneration was seen in the liver tissue of the model group, and no obvious abnormality was found in the other groups (The tissue in the visual field was evenly stained, the hepatic cords were neatly arranged, the cytoplasm of the hepatocytes was abundant, and the morphology and structure were normal), Fig. 6C.

## Discussion

Hypertension is the leading preventable risk factor for CVD, which often needs the use of long-term antihypertensive medication therapy to manage blood pressure within the recommended therapeutic range^1,26^. Most antihypertensive drugs still provide inadequate blood pressure control, with just 39% of hypertension patients meeting the therapeutic objective of blood pressure less than 140/90 mmHg^27^, so a more effective and safe strategy using antihypertensive drug combinations with western medicine are needed. In China, combining Chinese and western medicine is a common treatment regimen for treating a wide range of diseases^28^. The primary idea for treating hypertension in the guide to prevent and treat hypertension is combination treatment, which is thought to be useful for strengthening the antihypertensive impact without increasing undesirable effects^28^. The systematic review found that combining Chinese herbal medicine with western medicine reduced SBP and DBP to the target level in primary hypertensive patients better than western medicine alone, and no patients discontinued therapy or withdrew due to serious adverse effects, suggesting that combining the Chinese herbal medicine with western medication may be safe for hypertension treatment^29^. Captopril, an angiotensin-converting enzyme inhibitor, is extensively used in the treatment of hypertension and cardiovascular disease, and it also possesses antioxidant properties^30^. Previous research found that GJD may lower systolic and diastolic blood pressure in high salt fed rats^20^. In this study, GJD was used in conjunction with western medicine to treat spontaneously hypertensive rats to assess antihypertensive, vasoactive effects, anti-oxidant, aorta remodeling, and kidney protection. We found that 6 weeks of GJD administration combined with captopril treatment could significantly decrease both SBP and DBP better than captopril alone or GJD alone. Thus, our results revealed that GJD in combination with captopril can control blood pressure efficiently in SHRs. This result corresponds to the finding of several previous studies where combination therapy especially Chinese herbal formula with captopril a western medicine has a significant effect in decreasing blood pressure^31,32^.

Ang II has previously been shown to be significant in the control of vascular tone. It binds to the Ang II receptor type 1 (AGTR1) and works as a powerful vasoconstrictor, modulates aldosterone production, and remodels the heart and arteries, ultimately leading to hypertension and cardiac dysfunction^33^. Furthermore, hypertension and associated cardiovascular problems are caused by aberrant activation of the renin-angiotensin system (RAS). Similar to previous findings, SHR expresses various major RAS-related genes in the kidneys^34^ and has increased serum Ang II levels^35^. Previous studies indicated that the GJD might decrease Ang II level and AGTR1 expression^20,21^. According to our investigation, serum Ang II levels and AGTR1 protein expression in the thoracic aorta and kidney tissue were considerably lowered in all treatment groups, with the combination group experiencing the most profound reductions.

Endothelial cells are key elements of blood arteries that regulate blood fluidity and fibrinolysis, vascular tone, angiogenesis, monocyte/leukocyte adhesion, and platelet aggregation^36^. ET-1, a powerful vasoactive substance, plays an important role in controlling endothelial function and has a significant endogenous biological vasoconstrictive action^36,37^. The previous finding indicated that the GJD might affect the ET-1 level in hypertensive rats' serum^21^, and captopril decreased the ET-1 level in the kidney of SHR rats^31^. These results were in line with previous studies where the vasoconstrictor factor ET-1 level in serum and ET-1 expression in kidney and thoracic aorta was markedly decreased throughout all treatment groups, with the GJD + CAP group experiencing the greatest reductions.

Oxidative stress is a common cause of damage in many disease states, and it arises when the body's production of ROS exceeds its antioxidant defenses^38^. Since ROS and redox signaling play crucial roles in cardiovascular disease and renal impairments, oxidative stress is particularly relevant in hypertension^39^. Multiple studies have shown higher levels of ROS in primary hypertensive individuals and diverse animal models of this disease^40^. The fact that these patients and animals have a reduced antioxidant level supports the idea that enhanced vascular oxidative stress may have a role in the pathophysiology of primary hypertension^41–44^. In hypertension, ROS are produced by NADPH oxidase, xanthine oxidase, uncoupled eNOS, and mitochondria^45^, as well as Ang II and ET-1, which trigger transcription factors and redox-sensitive signaling pathways that alter vasculature function and structure^46^. Enzymatic antioxidants include SOD, GSH-Px, and CAT^47^. These antioxidants protect cellular lipids, proteins, and DNA from oxidative damage^48^. In our study, SOD activity was significantly lower in SHR hypertensive rats compared to WKY rats in the control group. These results are consistent with earlier research^12^. SOD is one of the most important antioxidant enzymes that convert the harmful superoxide anion O2 − %to harmless oxygen and hydrogen peroxide^49^. The toxicity of superoxide is reduced during this reaction and no free radicals are created^50^. In contrast to the SHR group, treatment groups receiving GJD, CAP, and GJD + CAP in this study showed a significant increase in SOD. A significant increase was also seen in the GJD + CAP group.

CAT and GSH-Px are two other antioxidant enzymes that detoxify free radicals by degrading H_2_O_2_ to oxygen and water^47^. GSH-Px may also diminish lipid peroxides and other organic hydroperoxides, which are very cytotoxic^47,51,52^. In the current research, the activity of CAT and GSH-Px enzymes decreased more significantly in the SHR group than in the WKY group, whereas it increased markedly in the GJD, CAP, and GJD + CAP groups compared to the SHR. The GJD + CAP group showed the most significant increase in CAT and GSH-Px levels. The SHR group in our research revealed a reduction in the production of antioxidative enzyme CAT and GSH-Px, which might be caused when the oxidative load exceeds the defense capability^53^. It is well known that antioxidant treatment increases CAT and GSH-Px activity, which was validated in the current investigation, with a more substantial increase reported in the combination group.

Furthermore, MDA is an oxidative product formed in the body by free radicals destroying fat, which may be utilized as an oxidative damage marker^54^. The findings of this investigation revealed that SHR rats have significantly increased levels of MDA than the WKY control group, which correlates to the findings of previous studies on oxidative stress in hypertensive rats^12,55^. As a result, rising MDA levels in hypertension indicate an increase in lipid peroxidation. In the current investigation, treatment of SHR rats with GJD, CAP, and the combination group GJD + CAP reduced MDA levels significantly, with the combination group showing the greatest decrease. This effect may be due to the GJD and captopril that neutralize the free radicals generated by lipid peroxidation^56,57^. Consequently, a decrease in MDA levels in all treatment groups and a greater reduction in the combination group may be attributable to the antioxidant impact of the combination GJD with captopril.

Nitric oxide is a transmitter produced by eNOS that plays an important role in blood vessel vasodilation^58,59^. NO bioavailability may decrease during hypertension due to decreased eNOS production or increased NO inactivation by oxidative stress^60^. Indeed, several studies indicate that oxidative stress is responsible for decreased NO bioavailability, which leads to reduced vasodilation and endothelial dysfunction in individuals with hypertension^61–63^. Furthermore, NO is required to regulate renal perfusion and glomerular filtration in the kidneys^60^. Consequently, Ang II induced oxidative stress reduces NO bioavailability in the renal microvasculature, leading to increased afferent arteriolar tone and hypertension^64,65^. In our study, the SHR group had considerably lower levels of NO and eNOS than the WKY control group. It has also been observed that endothelial dysfunction in SHR has been linked to elevated reactive oxygen species (ROS) generation in the vascular endothelium, notably superoxide anions, which are known to inactivate NO and thereby reduce NO bioavailability^66^. Indeed, ROS may oxidize eNOS cofactors such as tetrahydrobiopterin, causing the enzyme to switch from dimeric to monomeric form^67^. In the monomeric state, eNOS is uncoupled and superoxide anion is generated instead of NO, which may have negative repercussions and contribute to cardiovascular disorders, including hypertension^67^. In our study, NO serum level and eNOS expression in the aorta and kidney tissues increased significantly following treatment with GJD, CAP, and the combination group GJD + CAP, with the combination group showing the greatest increase, perhaps due to its strong anti-oxidative impact.

Increasing artery wall thickness is a common feature of hypertension-induced widespread vascular remodeling^68^. The previous study confirmed that GJD reduces smooth muscle layer thickness^21^, In the current study, we found that GJD combined with captopril treatment more significantly reduced vessel wall thickness.

The evaluation of biochemical markers reveals that medicinal herbs may have a negative impact on renal and hepatic function. Various biochemical indicators are important in detecting liver impairment and renal dysfunction of which ALT and AST are common liver enzymes that may be used to detect changes in liver function^69^, and screening tests for renal functioning are urea and creatinine^70^, and serum total protein analysis provides an estimate of nutritional status as well as a diagnostic assessment for changes in renal functioning^71^. Renal dysfunction causes inefficient urea and creatinine excretion, resulting in their buildup in the blood. Furthermore, histopathology of the kidney and liver tissues is regarded as an important technique for demonstrating hazardous substance-induced liver and kidney damage^72^. In this study, the kidney morphology changed significantly in SHR, however, the renal tissue damage biomarkers CREA, and TP did not change significantly. These findings were consistent with previous research^16^, which found that morphology changes in SHR rats were significantly presented at 12 weeks, but did not show a significant change in the CREA, BUN, and TP biomarkers until week 20, indicating that new sensitive biomarkers are required to detect early renal damage rather than old ones. In the current research, the histological investigation revealed modest congestive dilatation of glomerular capillaries in the SHR group, but the morphology and structure of the glomeruli were normal in all other groups. The previous research demonstrated that SHR develops congestive dilatation of glomerular capillaries, glomerular hypertension, ischemia with arteriolar constriction, and glomerular sclerosis and these changes begin to reverse following therapy^73^, this was validated in the current study. Therefore, according to our results, GJD extracts alone or in combination with captopril did not create liver or kidney toxicity in healthy rats, and it did not produce negative and/or toxic effects through histological and biochemical examination. This suggests that the drug's prescription is safe and has a protective effect on the kidney. However, more studies are necessary to clarify these results.

The results of the current study indicated that combination therapy by GJD + Captopril decreased blood pressure and the possible mechanism is via improving the serum, kidney, and thoracic aorta vasoactive factors by decreasing the vasoconstriction ET-1 and AngII and AGTR1 and the possible mechanism due to that the GJD effect^20,21^ with captopril^31,74^ which decrease these vasoconstriction factors. The increase in the vasodilation NO and eNOS may be due to the antioxidant effect of the GJD formula combined with the anti-oxidant effect of captopril that has a free radical scavenger activity^30,75,76^ that increases eNOS expression and reverses its uncoupling state and more synthesis of NO, finally, decrease blood pressure and tissue protection. Together, the combination of GJD with captopril was found to have a significant decrease in blood pressure, an anti-oxidant effect, and an influence on vasoactive factors that may improve aorta remodeling and kidney protection. These findings offer preliminary evidence for the possible future therapeutic use of GJD in combination with captopril as antihypertensive and renal protective effects.

In conclusion, the combination of GJD and captopril was found to significantly lower blood pressure, improve aorta remodeling, and kidney protection, possibly through an anti-oxidant effect (as measured by a decrease in MDA and an increase in SOD, GSH-Px, and CAT) and an influence on vasoactive factors (as measured by an increase in NO and eNOS and a reduction in ET-1, Ang II, and AGTR1). This in vivo study provides basic evidence for the potential future therapeutic use of GJD in combination with captopril as an antihypertensive.

## Materials and methods

### Materials

#### Preparation of GJD

The GJD, an aqueous ethanol extract containing six herbal medications (as presented in Table 2) were divided into two parts and soaked for half an hour in ethanol solution in a 1 g:10 ml ratio; part one (*Salvia miltiorrhiza* 25 g, *Pueraria lobate* 30 g, *Achyranthes bidentata* 20 g, *Eucommia ulmoides* 15 g, and *Prunella vulgaris* 15 g were decocted twice for one and a half hours in 60% ethanol, (during reflux, maintain the solution's boiling)) and part two (*Uncaria rhynchophylla* 10 g was mixed with 70% ethanol and decocted two times for two hours at 65 ~ 75 °C). Degreasing gauze was layered six times for each part. After that, soaked, reheated, and filtered the solution, then combined the two filtrates. The ethanol was then distilled at low pressure that used a rotary evaporator, followed by drying under low pressure and vacuum to give extract powder of the two parts. Finally, the two extract powder parts were mixed to produce GJD. High performance liquid chromatography (HPLC) was established to determine the active ingredients and it mainly contained puerarin (Purity: 2.31%), tanshinone IIA (Purity: 0.25%), daidzein (Purity: 0.5%), ursolic acid (Purity: 0.6%), and cryptotanshinone (Purity: 0.26%)^20^. The GJD botanical herbs dosage preparation processes follow the patent published (No. CN114246896A) and previously published articles^20,21^.

**Table 2:** Medications, kits, and devices used in this study.

Type	Name	Number	Company
GJD herbal medications	Uncaria rhynchophylla (Miq.) Miq. ex Havil 10 g	Lot No. 20200901	Heilongjiang Xiushengtang Pharmaceutical Co., Ltd.. Heilongjiang, China
	Salvia miltiorrhiza Bunge 25 g	Lot No. 20191001	
	Pueraria lobata (Willd.) Ohwi 30 g	Lot No. 20191001	
	Eucommia ulmoides Oliv., 15 g	Lot No. 20190901	
	Prunella vulgaris L., 15 g	Lot No. 20190701	
	Achyranthes bidentata Blume 20 g	Lot No. 20200901	
Western medicine	Captopril	Lot No. A0411A	Meilun Biotechnology Co., Ltd Dalian, China
Biochemical parameters	CRE	Lot No. 20210927	Rayto Life and Analytical Sciences Co., Ltd., Shenzhen
	BUN	Lot No. 20210630	
	TP	Lot No. 20210809	
	AST	Lot No. 20210726	
	ALT	Lot No. 20211008	
	DBIL	Lot No. 20210628	
	GGT	Lot No. 20210705	
	TBA	Lot No. 20211209	Jiancheng Bioengineering Institute, Nanjing
Elisa Kits	ET-1	Lot No. 20211217	Jiancheng Bioengineering Institute, Nanjing
	Ang II	Lot No. 20211217	
	NO	Lot No. 20211209	
	SOD	Lot No. 20211211	
	GSH-Px	Lot No. 20211208	
	MDA	Lot No. 20211210	
	CAT	Lot No. 20220903	
Blood pressure measurement devise	ALC-NIBP noninvasive BP system		Alcott Biotechnology Co., Ltd., Shanghai
Electronic balance	AL204electronic balance		Mettler-Toledo Measurement Co., Ltd., Changzhou
Microplate reader	M200pro		Tecan Austria GmbH, Austria
Automatic biochemical analyzer	Chemray 240		Rayto Life and Analytical Sciences Co., Ltd., Shenzhen, China

*GJD* Gedan Jiangya Decoction, *CRE* creatinine, *BUN* blood urea nitrogen, *TP* total protein, *AST* aspartate aminotransferase, *ALT* alanine aminotransferase, *DBIL* direct bilirubin, *GGT* γ-glutamyl transferase, *TBA* total bile acid, *ET-1* endothelin-1, *Ang II* angiotensin II, *NO* nitric oxide, *SOD* superoxide dismutases, *GSH-Px* glutathione peroxidase, *MDA* malondialdehyde, *CAT* catalase.

### Animal

The Vital River Laboratory Animal Technology Co. Ltd (Beijing, China) provided 32 SHR and 8 Wistar Kyoto (WKY) male rats aged 10 weeks (243 ± 10 g). License for experimental animals: SYXK(Hei)2018–007. All rats were kept in a pathogen-free laboratory animal room (temperature: 22 °C ± 2 °C; relative humidity: 53%-62%) with a 12-h light/dark cycle, and they were given standard feed and water ad libitum. The ethical permission was in accordance with standards for the protection of animals used in experiments (Directive 86/609/EEC), and the Animal Ethics Committee of Heilongjiang University of Chinese Medicine reviewed and approved the animal study (Approval No. 2020031203). All procedures were conducted in accordance with the ARRIVE guidelines.

### Groups and dosages

After a 10-day acclimatization period, 32 SHR were separated into four groups at random, each with eight rats; According to previous studies that utilized body surface area to convert dosages from humans to animals, the most effective dosage of GJD was selected^21^, as was the dose of captopril^77^. Group one (SHR): a hypertension model that was gavaged with 1.5 ml of 0.9% normal saline solution every day at 10 a.m. for 6 weeks. Group two (CAP): gavaged with captopril 13.5 mg/kg/d dissolved in 1.5 ml of 0.9% normal saline solution every day at 10 a.m. for 6 weeks. Group three (GJD): gavaged with GJD powder 5.44 g/kg/d dissolved in 1.5 ml of 0.9% normal saline solution every day at 10 a.m. for 6 weeks. Group four (GJD + CAP): a combination group that gavaged GJD powder 5.44 g/kg/d and captopril 13.5 mg/kg/d dissolved in 1.5 ml of 0.9% normal saline solution every day at 10 a.m. for 6 weeks. The normotensive control (WKY) was given a gavage with an equal volume of 0.9% normal saline solution every day at 10 a.m. for 6 weeks.

### Measurement of blood pressure and body weight

Body weight, SBP, and DBP were measured once a week. The blood pressure measuring protocol was created using the approach described in previous studies^78,79^. The ALC-NIBP, a non-invasive, computed tail-cuff blood pressure measurement and analysis device, was utilized to measure the SBP and DBP. Before the experiment, rats were acclimated to this procedure for one week. A cuff with a pneumatic pulse sensor was affixed to the tail. The ALC-NIBP system automatically measures blood pressure by monitoring the rat's arterial pulse signal with a very sensitive pulse sensor, blocking the arterial pulse with an inflated balloon, and detecting and analyzing changes in the pressure of the balloon and the pulse signal. The blood pressure value selection is based on the cuff pressure when blood flow is blocked, SBP is the pressure at the point when the pulse wave disappears, then automatically decompresses and DBP is the pressure at which the pulse wave begins to weaken. BP was measured first, followed by gavaging; three measures were conducted for each rat, and an average value was recorded. At the end of the experimental period, the rats were weighed accurately and anesthetized using sodium pentobarbital (50 mg/kg, i.p.). The serum, thoracic aorta, kidney, and liver tissue were collected for further analysis.

### Biochemical measurement

GJD's safety evolution was examined six weeks after treatment. Creatinine (CRE), blood urea nitrogen (BUN), total protein (TP), aspartate aminotransferase (AST), alanine aminotransferase (ALT), direct bilirubin (DBIL), total bile acid (TBA), and γ-glutamyl transferase (GGT) were all measured in the rats' serum. The kits for these parameters were provided by the Rayto Life and Analytical Sciences Co., Ltd., Shenzhen, and Jiancheng Bioengineering Institute in China, and all procedures were carried out in accordance with the instruction of reagent kits.

### ELISA Kits of vasoactive and oxidative stress parameters

All kits (as presented in Table 2) were obtained from the Jiancheng Bioengineering Institute, Nanjing, China, and were measured precisely according to the manufacturer's instructions.

For the determination of AngII and ET-1 levels, the same methods were used by adding samples to the enzyme-labeled wells pre-coated with antibodies, and then biotin-labeled recognition antigens were added and incubated at 37 °C for 30 min. The immune complex was formed and unconjugated biotin antigens were removed by PBST washing. Then, avidin horseradish peroxidase was added and incubated at 37 °C for 30 min. After washing, the combined horseradish peroxidase catalyzed tetramethylbenzidine to blue, which was then converted to yellow by adding the stopped solution of acid. The concentration of AngII and ET-1 was determined by measuring the absorbance at 450 nm. The SOD level was observed by the WST-1 method. SOD catalyzes the disproportionation reaction of the superoxide anion and inhibits the reaction of the superoxide anion produced by xanthine oxidase with WST-1 to produce methazine dye. The activity of SOD was calculated by measuring the absorbance of the reaction product at 450 nm. The MDA level was determined by the TBA method. A red product was obtained when MDA was reacted with thiobarbituric acid (TBA), exhibiting an absorbance peak at 532 nm.

The relatively stable yellow color results from the reaction of GSH with dithiodinitrobenzoic acid to create the 5-thiodinitrobenzoic acid anion was determined by measuring GSH-Px absorbance at 412 nm. The reaction of CAT to break down H_2_O_2_ was halted by adding ammonium molybdate to measure the CAT level, and the residual H_2_O_2_ interacted with ammonium molybdate to create a light-yellow complex. The absorbance was measured at 405 nm, and then the activity of CAT was calculated per milliliter of serum decomposed 1 μmol of H_2_O_2_ per second into an activity unit (U).

### qRT-PCR of AGTR1, eNOS, and ET-1

Total RNA was isolated from kidney and thoracic aorta tissues using the Trizol reagent (Takara). For cDNA synthesis, a PrimeScript RT Kit (Takara) was employed. We used TB Green® Premix Ex TaqTM II (Tli RNaseH Plus) in a real-time polymerase chain reaction assay (Takara). The QuantStudioTM3 Real-time PCR technology was used to quantify gene expression levels. The 2-ΔΔCT approach was utilized to analyze and evaluate the data, with GAPDH as a reference gene to calculate and interpret the results. The primer sequences utilized in qRT-PCR are listed in Table 3.

**Table 3:** qRT-PCR primer sequences.

Gene	Forward (5′–3′)	Reverse (5′–3′)
AGTR1	CATGATCCCTACCCTCTACAGC	TAAATGACAATCACCACCAAGC
ET-1	GTCTAAGCGATCCTTGAA	AATTCCAGCACTTCTTGT
eNOS	GGATTCTGGCAAGACCGATTAC	GGTGAGGACTTGTCCAAACACT
GAPDH	TGCACCACCAACTGCTTAG	GATGCAGGGATGATGTTC

### HE staining and Immunohistochemistry

The aorta, kidney, and liver tissues were removed fresh and then fixated with 4% paraformaldehyde at 4° C for 24 h before dehydrating. Fixed tissue was dried, embedded in paraffin, sectioned to a thickness of 5 microns, and stained with hematoxylin–eosin (H&E). A Nikon Eclipse Ci-L (Japan) optical microscope was used to pick the target location of the tissue and to fill the entire visual field with tissue. Each slice had at least three 200^×^ visual fields taken at random and inspected under a microscope. Two veterinary pathologists evaluated HE-stained slides to identify abnormalities. For immunohistochemistry, tissue sections of the thoracic aorta and kidney were deparaffinated in xylene and dehydrated with a series of alcohol. The slices were treated in 3% H2O2 at room temperature for 25 min in the dark to inhibit endogenous peroxidase activity, then rinsed with phosphate-buffered saline (PBS). After 30 min of blocking at room temperature, sections were treated with diluted primary antibodies at 4° C for one night. (AGTR1, ET-1, and eNOS). The slices were then washed in phosphate-buffered saline and incubated for 50 min with the appropriate secondary antibodies. After that, the slices were stained with 3,3-diaminobenzidine and counterstained with hematoxylin (DAB, Servicebio, China). The sections were dehydrated by immersing them in a sequence of solvents until they became transparent and dehydrated. The parts of sections were then mounted with SweSuper Clean BioMount Medium. The primary antibodies utilized for detecting were against the AGTR1 (GB112004, 1:500, Servicebio, China), ET-1 (bs-0954R, 1:500, Bioss, China), and eNOS (GB12086, 1:500, Servicebio, China). The target region of the tissue was selected at random using the light microscope Eclipse Ci-L with a magnification of 200x. Using the pixel area as the reference, values for integrated optical density (IOD) taken from each of the three different slices of the visual field were calculated using the image analysis program Image Pro Plus 6.0.

### Statistical analysis

All data is displayed as mean ± standard deviation (Mean ± SD), and it was statistically evaluated using the GraphPad Prism 7.0 software. One-way ANOVA with Tukey's multiple comparisons was used to analyze statistical differences. P values less than 0.05 were considered as statistically significant.

## Supplementary Information

Supplementary Information.

## Data Availability

The data used to support the findings of this study are included within the supplementary material.

## Abbreviations

GJD: Gedan Jiangya Decoction
SBP: Systolic blood pressure
DBP: Diastolic blood pressure
CVD: Cardiovascular disease
SHR: Spontaneously hypertensive rats
WKY: Wistar Kyoto rats
NO: Nitric oxide
SOD: Superoxide dismutases
GSH-Px: Glutathione peroxidase
CAT: Catalase
MDA: Malondialdehyde
ET-1: Endothelin-1
Ang II: Angiotensin II
AGTR1: Ang II receptor type 1
eNOS: Endothelial nitric oxide synthase
CRE: Creatinine
BUN: Blood urea nitrogen
TP: Total protein
AST: Aspartate aminotransferase
ALT: Alanine aminotransferase
DBIL: Direct bilirubin
TBA: Total bile acid
GGT: γ-Glutamyl transferase
ROS: Reactive oxygen species
